# Supplementary material for: Complex‐centric proteome profiling by SEC‐SWATH‐MS
Source: Mol Syst Biol. 2019 Jan 14;15(1):e8438. doi: 10.15252/msb.20188438 (PMC6346213; doi:10.15252/msb.20188438)
Supplement: Supplementary file 8 — Dataset EV7 [file MSB-15-e8438-s008.zip › feature_plots_string/Q9NQZ2.pdf]

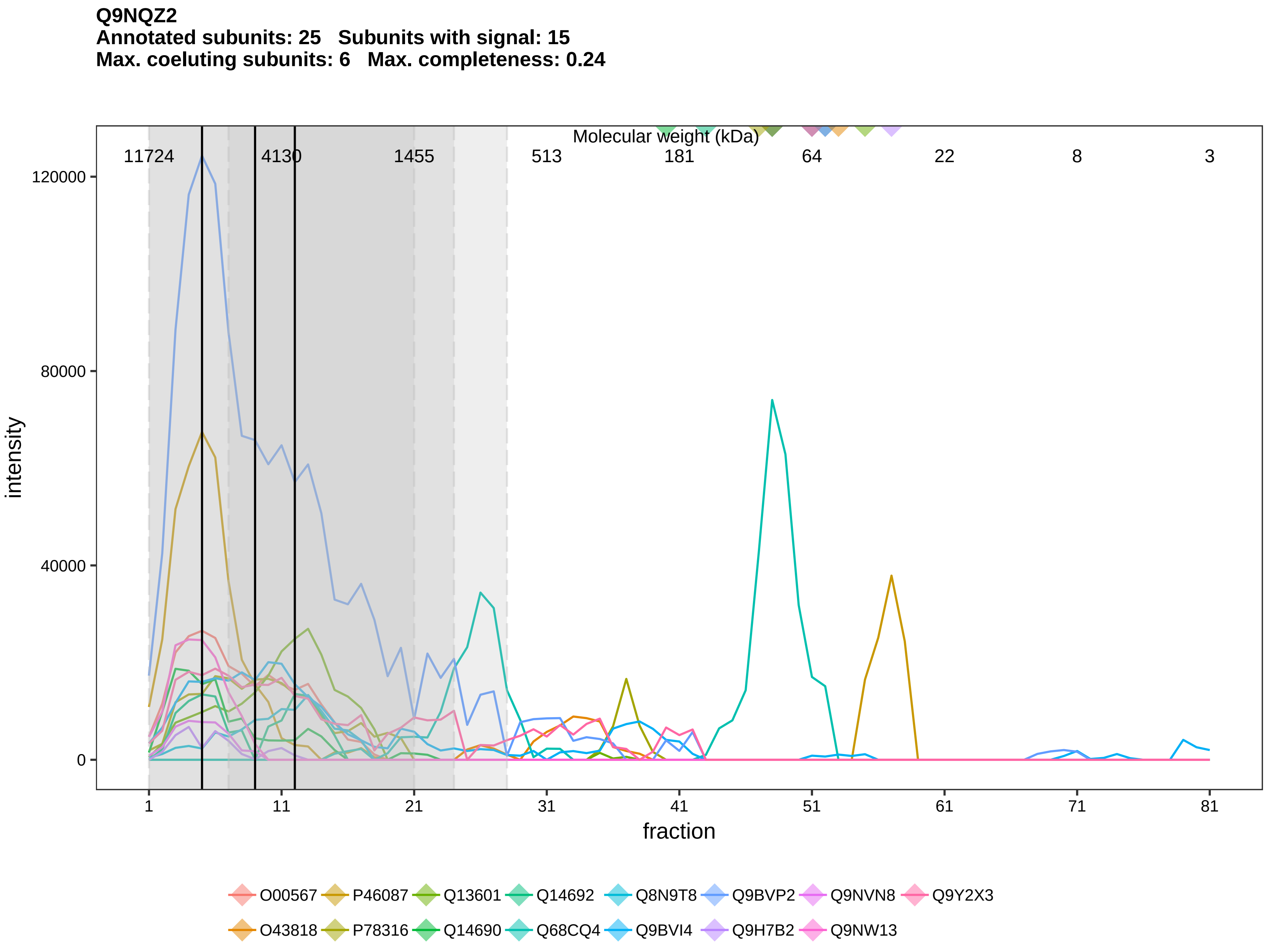
Q9NQZ2
Annotated subunits: 25 Subunits with signal: 15
Max. coeluting subunits: 6 Max. completeness: 0.24
Molecular weight (kDa)
11724
4130
1455
513
181
64
22
8
3
120000
80000
40000
0
intensity
1
11
21
31
41
51
61
71
81
fraction
O00567
P46087
Q13601
Q14692
Q8N9T8
Q9BVP2
Q9NVN8
Q9Y2X3
O43818
P78316
Q14690
Q68CQ4
Q9BVI4
Q9H7B2
Q9NW13
